# Problem of Caring for the Premature Baby

**Published:** 1913-11

**Authors:** 


PROBLEM OF CARING FOR THE PREMATURE BABY. Bulletin of the Lying-in Hospital of the city of New York, November, 1912. Coolidge emphasizes the importance of: 1. Feeding, if necessary, by gauge. 2. Not mistaking apathetic nursing for satiety. 3. Anything but the incubator, the best plan being the incubator room as conducted at the Babies' Hospital. Room is kept at 90° F. with constant ventilation, constant and painstaking care to prevent chilling, irritation or infection of the skin.

The Mammary Glands and Eclampsia: Report of a case treated with Oxygen Infiltration of the Breasts.

James A. Harrar, Att. Surgeon, Sellheim, in 1910, called attention to apparent elaboration of toxin in breasts of eclamptics and went so far as to amputate for this condition.

Goodall, in 1911, reported three cases of infants apparently poisoned by eclamptic mother's milk.

In March, 1912, Jr. of Infect. Diseases, Kastle & Healy, on basis of successful treatment of parturient paresis in the cow, recommended the distention of the submammary areolar tissues with Oxygen for eclampsia.

Gilles and Ducuing reported the recovery of a case following this procedure, likewise E. Williams. Finally the writer reports another case in point. The injection was followed by no serious results but by a rather alarming spread of the artificial emphysena all over the upper body. In a week the patient had recovered from the disease as well as its treatment.

